# Supplementary material for: Analysis of PPARγ Signaling Activity in Psoriasis
Source: Int J Mol Sci. 2021 Aug 10;22(16):8603. doi: 10.3390/ijms22168603 (PMC8395241; doi:10.3390/ijms22168603)
Supplement: Supplementary file 1 [file ijms-22-08603-s001.zip › Supplemental materials_Analysis of PPARg signaling activity in psoriasis/Pathway models/Models images and html files/Model of PPARG signaling in psoriasis/142791.html]

lipid metabolism


# Cell Process lipid metabolism

|  |  |
| --- | --- |
| URN | urn:agi-gocellproc:0006629 |
| Total Entities | 20 |
| Connectivity | 4515 |
| Name | lipid metabolism |

---

|  |  |
| --- | --- |
| ChildConcepts | lipid homeostasis |
|  | lipid oxidation |
|  | lipid degradation |
|  | oligosaccharide-lipid intermediate assembly |
|  | GPI anchor metabolism |
|  | fatty acid metabolism |
|  | lipogenesis |
|  | fatty acid desaturation |
|  | phospholipid biosynthesis |
|  | steroidogenesis |
|  | sterol metabolism |
|  | sterol biosynthesis |
|  | galactolipid biosynthesis |
|  | neutral lipid biosynthesis |
|  | lipid modification |
|  | lipid glycosylation |
|  | GSI anchor metabolism |
|  | membrane lipid biosynthesis |
|  | lipid peroxidation |
|  | lipid hydrolysis |

---

|  |  |
| --- | --- |
| Pathway | Estrogen Deficiency in Female Obesity |
|  | Androgen Deficiency in Male Obesity |
|  | mTOR Signaling |
|  | AMPK Related Catabolism Deceleration in Glucose Insufficiency |
|  | Thyroid Hormones Common Genomic Effects in Hyperthyroidism |
|  | Insulin Signaling |
|  | Androgens in Adipocyte Activation |
|  | Adipokines Production by Adipocyte |
|  | Familial Partial Lipodystrophy Type 4 Progression (Hypothesis) |
|  | Donohue Syndrome Progression (Hypothesis) |
|  | Familial Partial Lipodystrophy Type 3 Progression (Hypothesis) |
|  | Familial Partial Lipodystrophy Type 2 Progression (Hypothesis) |
|  | Berardinelli-Seip Syndrome Progression (Hypothesis) |
|  | Glucocorticoids in Insulin Resistance in Skeletal Muscles |
|  | Adipokines and Cytokines in Insulin Resistance in Skeletal Muscles |
|  | Adipokines Production by Adipocyte Impaired in Obesity |
|  | Peutz-Jeghers Syndrome |
|  | Lipodystrophy, Familial Partial |
|  | AMPK Signaling |
|  | Model of PPARG signaling in psoriasis |
|  | 4\_1\_Insulin resistance: insulin signaling |
|  | 2\_2\_Effects of thyroid hormone deficiency: cell-specific effects of thyroid hormone action |

---

|  |  |
| --- | --- |
| MedScan ID | 4106629 |

---

|  |  |
| --- | --- |
| Alias | turnover of lipid |
|  | lipometabolisms |
|  | lipid droplets turnover |
|  | lipometabolism |
|  | lipid metabolisms |
|  | lipid droplets metabolism |
|  | metabolism of fat |
|  | metabolism of lipid droplets |
|  | metabolism of lipid droplet |
|  | metabolisms of lipid |
|  | lipid mobilizations |
|  | lipid turnover |
|  | metabolism of lipid |
|  | lipid mobilization |
|  | lipid droplet metabolism |
|  | turnover of lipid droplets |
|  | fat metabolism |
|  | lipid droplet turnover |
|  | mobilization of lipid |
|  | fat metabolisms |
|  | turnover of lipid droplet |
|  | lipid metabolism |
|  | lipid mobilisations |
|  | lipid mobilisation |

---

|  |  |
| --- | --- |
| GO ID | 0006629 |

---
